# Sepsis and Pleural Empyema Caused by *Streptococcus pyogenes* after Influenza A Virus Infection

**DOI:** 10.1155/2018/4509847

**Published:** 2018-09-23

**Authors:** Fumihiro Ochi, Hisamichi Tauchi, Toshihiro Jogamoto, Hiromitsu Miura, Tomozo Moritani, Kozo Nagai, Eiichi Ishii

**Affiliations:** Department of Pediatrics, Ehime University Graduate School of Medicine, Ehime, Toon, Japan

## Abstract

*Streptococcus pyogenes* (also referred to as group A streptococci, GAS) causes severe invasive diseases such as bacteremia, necrotizing fasciitis, pneumonia, osteomyelitis, septic arthritis, and toxic shock syndrome in children. However, there are only a few reports on pleural empyema caused by GAS in children. Here, we report the case of a 4-year-old boy who presented with pleural empyema due to GAS after influenza A virus infection. With intravenous antibiotic administration and continuous chest-tube drainage, followed by video-assisted thoracoscopic surgery, his condition improved. During the clinical course, cytokines induced in response to the influenza virus, especially IL-1*β* and IL-10, were elevated 1 week after influenza A infection, but these decreased as the symptoms improved. Reportedly, the IL-10 production increases during influenza virus-bacteria superinfection. These observations suggest that the immunological mechanisms induced by the influenza virus can play an important role in influencing the susceptibility to secondary bacterial infections, such as GAS, in children.

## 1. Introduction

Lancefield group A beta-hemolytic streptococcus (GAS, *Streptococcus pyogenes*) causes a variety of infectious diseases in children; these range from common acute infections such as pharyngitis and impetigo to severe invasive diseases such as bacteremia, necrotizing fasciitis, pneumonia, osteomyelitis, septic arthritis, and toxic shock syndrome [1, 2]. However, only few reports describing pleural empyema due to GAS in children are available. For example, Krenke et al. [3] reported that bacteria detected by culturing the pleural fluid in children with parapneumonic effusion/pleural empyema were mainly *Streptococcus pneumoniae* (66.7%) or coagulase negative staphylococci (14.7%) and only a small proportion of GAS (5.8%).

Here, we report the case of a 4-year-old boy with pleural empyema due to GAS after infection with influenza A virus. In addition, we discuss the pathogenesis of severe GAS infection following influenza with regard to cytokine levels during the clinical course of GAS infection.

## 2. Case Presentation

**Figure 1:**
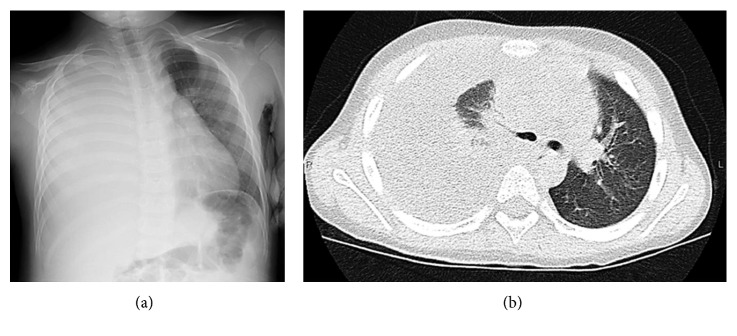
(a) Chest X-ray and (b) chest computed tomography scan of the thoracic region. The images indicate a large pleural empyema of a right pulmonary lesion.

A 4-year-old boy was treated for fever, mild cough, and nasal discharge at another hospital. Thereafter, he was diagnosed with influenza A infection and was treated with oseltamivir (4 mg/kg/day, 5 days). However, his condition deteriorated and fatigue, low activity, and breathing difficulty progressed; he was admitted to our hospital 7 days after the diagnosis of influenza A infection. He had no medical history of recurrent bacterial infections or growth failure. Physical examination at admission revealed remarkable respiratory distress and consciousness disturbance (Glasgow Coma Scale, E4V3M4). His body temperature was 38.0°C, blood pressure was 126/77 mmHg, heart rate was 155 beats/min, respiratory rate was 60 breaths/min, and oxygen saturation was 90% at room air. Right breath sounds were reduced, and an end-inspiratory crackle was detected in the right upper lung. In addition, red, cracked lips, strawberry tongue, and trunk and bilateral feet erythema were observed. Neither bilateral conjunctival injection, cervical lymphadenopathy nor edema was detected. Laboratory examination revealed a white blood cell count (WBC) of 20,000/*μ*L with 95.1% neutrophils, hemoglobin level of 14.0 g/dL, and platelet count of 22.7 × 10^4^/*μ*L. Inflammatory biomarkers were elevated; C-reactive protein level was 20.54 mg/dl, procalcitonin level was 45.23 ng/mL, lactate dehydrogenase (LDH) level was 512 U/L, ferritin level was 261 ng/mL, and soluble interleukin-2 receptor (sIL-2R) level was 6,176 U/mL. The levels of several cytokines were also increased: IL-1*β* was 1.3 pg/mL; IL-6 was 233 pg/mL; IL-10 was 67 mg/mL; and TNF-*α* was 2.6 pg/mL, whereas IL-2, IL-3, IL-4, IL-5, and IL-12 were all normal. A chest X-ray and chest computed tomography revealed consolidation and a large-right pleural effusion (Figures 1(a) and 1(b)). Based on these findings, the patient was diagnosed with sepsis and pleural empyema due to GAS infection.

**Figure 2:**
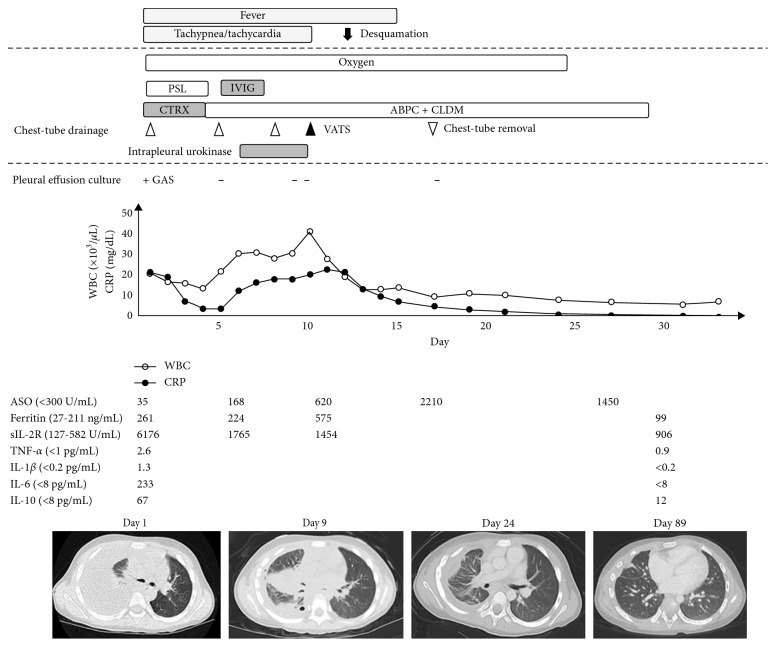
Clinical course of the patient. IL-1*β*, IL-6, IL-10, and TNF-*α* were elevated 1 week after the influenza infection but normalized as the symptoms improved. GAS, *Streptococcus pyogenes*; PSL, prednisolone; sIL-2R, soluble interleukin-2 receptor; ASO, antistreptolysin O antibody; WBC, white blood cell counts; CRP; C-reactive protein, CTRX, ceftriaxone; ABPC, ampicillin; CLDM, clindamycin; IVIG, immunoglobulin; and VATS, video-assisted thoracoscopic surgery.

**Figure 3:**
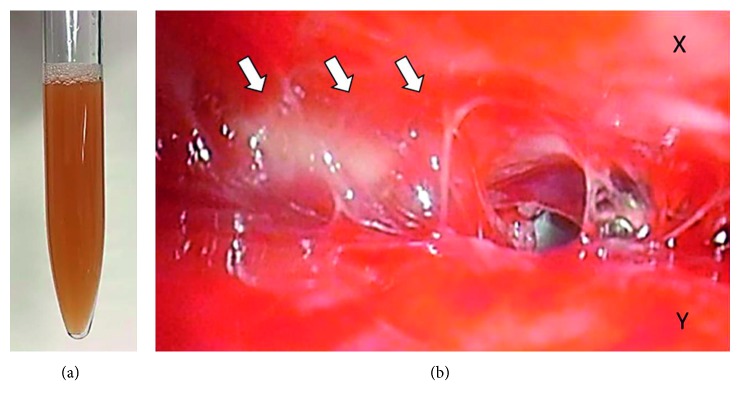
(a) Aspirated pus from the pleural empyema. (b) Operative thoracoscopic views obtained from video-assisted thoracoscopic surgery. White arrows indicate severe adhesion by fibrin between the parietal pleura (X) and the pulmonary pleura (Y).

The clinical course of the patient is shown in Figure 2. Initially, continuous chest-tube drainage, intravenous administration of antibiotics (CTRX) and immunoglobulin (150 mg/kg/day, 3 days), and prednisolone (1 mg/kg/day, 3 days) were prescribed for sepsis and pleural empyema. The aspirated pleural fluid was serological with a yellowish brown color and pH of 7.002, WBC of 33,900/*µ*L, protein of 4.7 g/dL, LDH of 9,121 U/L, and adenosine deaminase of 173.7 U/L (Figure 3(a)). No bacteria were detected by blood culture, whereas GAS tests conducted on both pleural fluids and throat swab on day 1 after culture were positive. The genotype of GAS was found to be *emm1*/*speA*/*speB*/*speF*, which is known as a virulence factor gene. Based on this data, the antibiotics were changed from CTRX to ampicillin (ABPC) and clindamycin (CLDM). However, fever and tachypnea persisted. Alternatively, the chest tube was considered as being obstructed or failing to drain, and hence, the chest tube was replaced, and an additional fibrinolytic therapy (urokinase 40,000 units in 40 mL 0.9 percent saline, intrapleural) was included; however, the patient's clinical condition still did not improve. On day 9, video-assisted thoracoscopic surgery (VATS) was performed to remove the thick fibrous septations. Thoracoscopy of the thoracic cavity revealed an adhered pulmonary and parietal pleura with fibrin; the cavity was then peeled off and washed using saline (Figure 3(b)). The patient's clinical condition improved following VATS, and he was discharged on day 33. All inflammatory biomarkers and cytokines had reduced to normal levels. At the time of the final follow-up, the patient had been healthy without any symptoms.

## 3. Discussion

GAS is a well-known causative pathogen of the upper respiratory tract and cutaneous infections and occasionally leads to parapneumonic effusion/pleural empyema. Sakai et al. described an invasive GAS infection with community-acquired pneumonia followed by pleural empyema that resulted in streptococcal toxic shock syndrome in a healthy male adult [2].

GAS strains express many bacterial virulence factors including protein M, streptolysins, and streptokinase. Of these, protein M is a major bacterial virulence factor that is encoded by the *emm* gene and shows resistance to phagocytosis. The *emm1* gene was most prevalent (*n*=27, 32.9%) and significantly related to poor outcomes among 82 invasive GAS isolates in Japan [4]. Furthermore, these invasive strains showed a highly significant association with the presence of the *speA* gene, which encodes the streptococcal superantigen [5]. Compatible with these findings, our isolate had *emm1*/*speA* genes, which is suggestive of a highly pathogenic strain.

Over recent years, the frequency of parapneumonic effusion/pleural empyema has increased in children with influenza A infection [6]. Furthermore, recent studies have attempted to elucidate the immunological mechanism of influenza virus complicated by bacterial superinfection. The secondary bacterial superinfection vulnerability typically surfaces 1 week after influenza infection [7]. In our case, the patient also incurred sepsis and pleural empyema as a result of GAS infection that surfaced 7 days after the onset of influenza A infection. These findings indicated the importance of immune status, particularly one week after influenza.

Robinson et al. [8] reported that influenza A virus inhibits bacteria-induced IL-1*β* production and impairs host defense against bacterial infection. Moreover, IL-10 deactivates macrophages and decreases the production of cytokines by T cells [9]; regulatory T (T_Reg_) cells are also known to produce IL-10 during influenza virus infection [10]. As the production of the anti-inflammatory cytokine IL-10 has been shown to increase during influenza virus-bacterial superinfection, it is possible that T_Reg_ cells induce susceptibility to secondary bacterial infection [11]. Barthelemy et al. [12] reported that influenza A virus-induced release of IL-10 inhibited the antimicrobial activities of invariant natural killer T cells during invasive pneumococcal superinfection. In addition, neutralization of IL-10 with a specific antibody reduced mortality and *S. pneumoniae* growth in mice [13]. Interestingly, recent evidence has suggested that the serum IL-10 level is high in cases of rheumatic fever (RF) or rheumatic heart disease (RHD) caused by GAS, and IL-10 plays a vital role in the pathogenesis and contributes to the severity of RF/RHD [14]. These findings emphasized the importance of IL-10 in influenza virus-bacterial superinfection.

Our patient exhibited elevated levels of serum IL-1*β* and IL-10 after one week of influenza A infection, but they decreased as the symptoms improved. It is notable that the increased level of IL-10 was remarkable compared with that of IL-1*β*, suggesting that cytokines induced by the influenza virus play an important role in the immunological mechanisms underlying influenza virus-GAS superinfection.

In conclusion, any immunological mechanisms induced by influenza virus play an important role in the susceptibility of secondary bacterial infections, such as GAS, in children.
